# Robotic Intracellular Pressure Measurement Using Micropipette Electrode

**DOI:** 10.3390/s23104973

**Published:** 2023-05-22

**Authors:** Minghui Li, Jinyu Qiu, Ruimin Li, Yuzhu Liu, Yue Du, Yaowei Liu, Mingzhu Sun, Xin Zhao, Qili Zhao

**Affiliations:** 1 Institute of Robotics and Automatic Information System, Tianjin Key Laboratory of Intelligent Robotics, Nankai University, Tianjin 300350, China; 2120210388@mail.nankai.edu.cn (M.L.); qiujinyu@mail.nankai.edu.cn (J.Q.); lrumin@mail.nankai.edu.cn (R.L.); liuyuzhu@mail.nankai.edu.cn (Y.L.); duy@nankai.edu.cn (Y.D.); liuyaowei@mail.nankai.edu.cn (Y.L.); sunmz@nankai.edu.cn (M.S.); zhaoxin@nankai.edu.cn (X.Z.); 2 Institute of Intelligence Technology and Robotic Systems, Shenzhen Research Institute of Nankai University, Shenzhen 518083, China; 3 Beijing Advanced Innovation Center for Intelligent Robots and Systems, Beijing Institute of Technology, Beijing 100081, China

**Keywords:** intracellular pressure, electrode circuit model, micropipette electrode, micro-nano operation

## Abstract

Intracellular pressure, a key physical parameter of the intracellular environment, has been found to regulate multiple cell physiological activities and impact cell micromanipulation results. The intracellular pressure may reveal the mechanism of these cells’ physiological activities or improve the micro-manipulation accuracy for cells. The involvement of specialized and expensive devices and the significant damage to cell viability that the current intracellular pressure measurement methods cause significantly limit their wide applications. This paper proposes a robotic intracellular pressure measurement method using a traditional micropipette electrode system setup. First, the measured resistance of the micropipette inside the culture medium is modeled to analyze its variation trend when the pressure inside the micropipette increases. Then, the concentration of KCl solution filled inside the micropipette electrode that is suitable for intracellular pressure measurement is determined according to the tested electrode resistance–pressure relationship; 1 mol/L KCl solution is our final choice. Further, the measurement resistance of the micropipette electrode inside the cell is modeled to measure the intracellular pressure through the difference in key pressure before and after the release of the intracellular pressure. Based on the above work, a robotic measurement procedure of the intracellular pressure is established based on a traditional micropipette electrode system. The experimental results on porcine oocytes demonstrate that the proposed method can operate on cells at an average speed of 20~40 cells/day with measurement efficiency comparable to the related work. The average repeated error of the relationship between the measured electrode resistance and the pressure inside the micropipette electrode is less than 5%, and no observable intracellular pressure leakage was found during the measurement process, both guaranteeing the measurement accuracy of intracellular pressure. The measured results of the porcine oocytes are in accordance with those reported in related work. Moreover, a 90% survival rate of operated oocytes was obtained after measurement, proving limited damage to cell viability. Our method does not rely on expensive instruments and is conducive to promotion in daily laboratories.

## 1. Introduction

Cells are able to maintain positive intracellular pressure compared to the extracellular environment. Intracellular pressure is an important component of the intracellular environment, which ensures the normal physiological function of cells. It has been found to play an important role in cell division [1,2], differentiation [3,4], migration [5], diseases [6,7], and embryonic tissue development [8,9]. Moreover, the existence of intracellular pressure affects the results of cell micromanipulation. For example, during cell microinjection, the positive intracellular pressure increases the resistance to the material delivery, resulting in lower deposition volume [10,11]. Furthermore, intracellular pressure supports cell swelling, making the cell membrane more resistant to external pressure. This can affect the measurement results of cell elasticity. Therefore, intracellular pressure measurement is beneficial for revealing the mechanism of cells’ physiological activities and improving the accuracy of cell micromanipulation.

At present, some methods have been developed to measure the intracellular pressure of different types of cells, according to references. For example, according to Laplace’s law, intracellular pressure has been estimated indirectly from the cortical tension of cells [12,13]. However, assuming the cell to be a liquid ball makes that method applicable to cells with thin membranes. For oocytes or embryos with thick zona pellucid (ZP), the measurement errors of that method may be too large to be applicable. Before and after the release of intracellular pressure caused by cutting off part of the ZP [14], the variations in the obtained poking force–cell deformation curves have been utilized to measure the intracellular pressure. Although this method is applicable for oocytes or embryos with thick ZPs, it uses laser cutting to release intracellular pressure and microforce sensors to obtain the poking force–cell deformation curves, which requires multiple, expensive pieces of equipment, significantly limiting its application. In previous research, we proposed an intracellular pressure measurement method based on the modeled relationship between the applied pressure and the deposition volume of oil into cells [11]. Although this method can be realized on a traditional cell manipulation system setup, the oil droplets injected into the cells cause significant damage to cell viability, limiting its application. Commercial intracellular pressure measurement devices with pressure feedback have also been used to measure intracellular pressure [15,16,17]. Although this system can measure the intracellular pressure of a variety of cells with limited harm to cell viability, the measurement device is very expensive, thus, significantly limiting its widespread use. Therefore, a simple intracellular pressure measurement method that is applicable to traditional cell manipulation tools and causes less harm to cell viability is still highly desired.

This paper proposes a robotic intracellular pressure measurement method using a traditional micropipette electrode. First, the measured resistance of the micropipette electrode filled with KCl solution and immersed inside the culture medium is modeled to analyze its variation trend when the pressure inside the micropipette increases. Then, the concentration of KCl solution producing a “steep slope” in the electrode resistance–pressure curve is determined as the one suitable for intracellular pressure measurement. Further, the measured resistance of the micropipette electrode inside the cell is modeled to obtain the intracellular pressure. Intracellular pressure is measured by the difference between two key pressures, which correspond to injection pressures *P*_I1_ and *P*_I2,_ when the micropipette electrode resistance reaches a quasi-stable state before and after the release of intracellular pressure. Based on the above work, a robotic measurement procedure of the intracellular pressure is established based on a traditional micropipette electrode system. After two weeks of continuous practice, scholars are able to successfully measure approximately 10 cells per day using commercial equipment. An experienced expert should expect to be able to measure 50–100 cells per day [15]. The experimental results on porcine oocytes demonstrate that the proposed method can operate on cells at an average speed of 20~40 cells/day with measurement efficiency comparable to commercial measurement devices. The average repeated error of the relationship between the measured electrode resistance and the pressure inside the micropipette electrode is less than 5%, and no observable intracellular pressure leakage was found during the measurement process, which guarantees the measurement accuracy of intracellular pressure. The measured results of the porcine oocytes are in accordance with those reported in related work. Moreover, a 90% survival rate of operated oocytes was obtained after measurement, proving limited damage to cell viability.

## 2. Materials and Methods

### 2.1. System Setup

**Figure 1:**
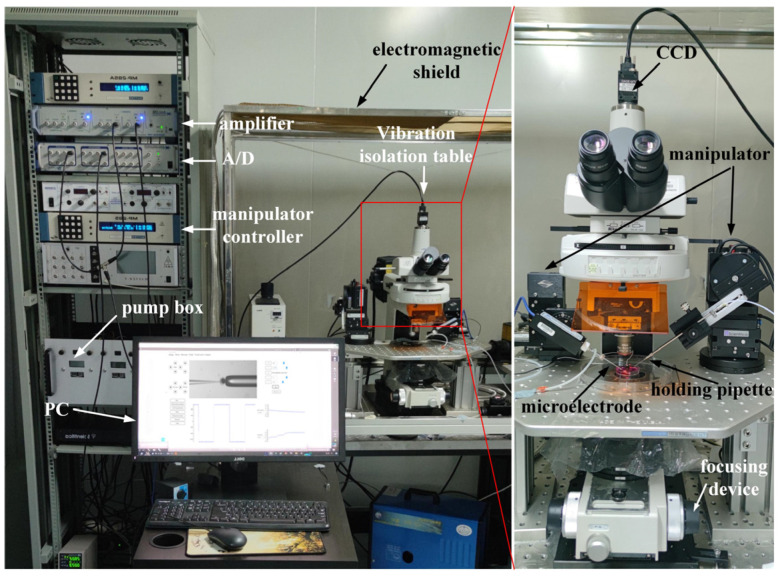
System setup.

**Figure 2:**
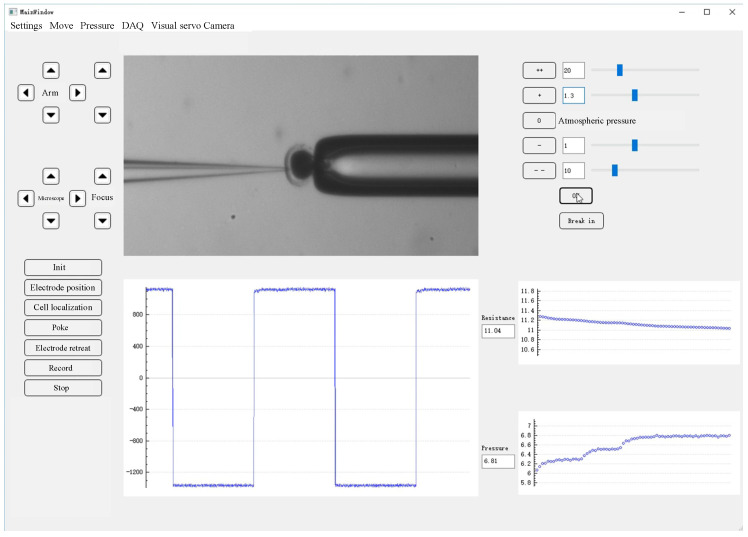
Human–machine interface.

We conducted the intracellular pressure measurement experiment on the micropipette electrode system developed in our laboratory. The system setting is shown in Figure 1. The 35 × 10 mm culture dish was placed on a fixed platform on the vibration isolation platform, in which the cell operating fluid and experimental samples were placed. A standard upright microscope (Eclipse FN1, Nikon, Tokyo, Japan) capable of movement in the XY plane with a repeatability of ±0.1 μm over an area of 2 × 2 cm^2^ was used to observe cells in the intracellular pressure measurement experiment. The motorized focusing device on the microscope was used to automatically focus the cells with a repeatability of ±0.1 μm in the vertical direction. On the left arm was an X-Y-Z micromanipulator model MP285 (stroke space 2 × 2 × 2 cm^3^, maximum speed 1 mm/s, repeatability ±0.1 μm) for mounting micropipette electrodes. The right arm was a scientific model X-Y-Z micromanipulator (stroke space 2.5 × 2.5 × 2.5 cm^3^) for mounting the holding pipette (HP). An in-house developed pneumatic chamber [18] was used to provide pressure inside the micropipette. A charge-coupled device (CCD) camera (IR-2000, DAGE-MTI, Michigan City, IN, USA) was mounted on the microscope to acquire images of the cells at 60 frames per second. A host computer was used for microscopic image processing, electrical signal acquisition, suction pressure control, and motion control of the microscope and manipulator. The entire robot system was covered with an electromagnetic shield to isolate electrical interference from the external environment. A human–machine interface based on Qt programming (see Figure 2) was developed to provide visual feedback and display information about the process of measuring intracellular pressure, suction pressure, electrical signals, output pressure, etc. This interface allows the operator to update the pressure applied inside the micropipette and the distance the micropipette electrode moves at each step. The electrical signal detected by the micropipette electrode was amplified using an amplifier (MultiClamp 700B, Molecular Devices, San Jose, CA, USA), then converted into a digital signal, and finally transmitted to the host computer.

### 2.2. Fabrication of Micropipette Electrode and Oocyte Preparation for Intracellular Pressure Measurement

**Figure 3:**
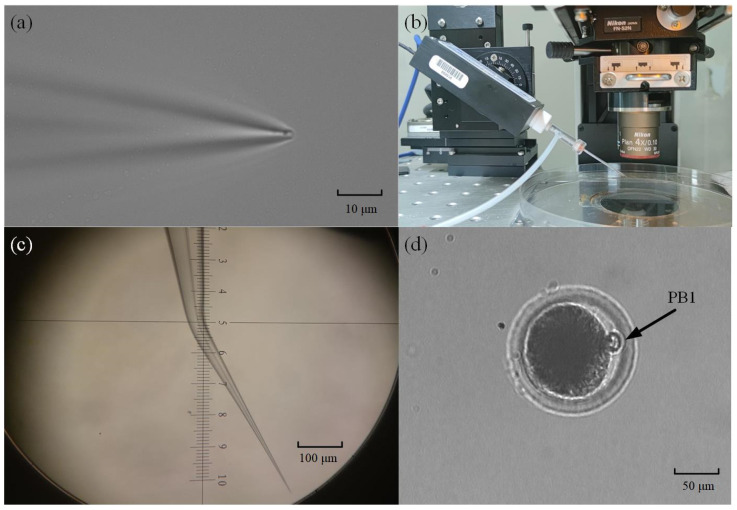
Fabrication of micropipette electrode and oocyte preparation: (**a**) Imaging of a drawn micropipette under a 40× microscope; (**b**) The angle between the micropipette electrode and the horizontal plane is 30°; (**c**) The micropipette is bent on the forging needle instrument; (**d**) Porcine oocytes with first polar body.

The micropipette electrode used in the intracellular pressure measurement experiment was made of a glass tube (BF150-117-10, Sutter, Sacramento, CA, USA) with an inner diameter of 1.17 mm and an outer diameter of 1.5 mm. The glass tube was pulled with a micropuller (P97, Sutter) to form a tapered tip with a diameter of ~2 μm (see Figure 3a). If that micropipette were mounted directly on the robot arm, the micropipette electrode would penetrate the cell at an oblique 30°. This is because the electrode holder on the robot is installed at an inclination of 30° (see Figure 3b) in consideration of the use limitation of space, the stroke of the robot, etc. Therefore, to reduce the torsional effect of micropipette penetration on cells, we used a micropipette forging instrument to bend the tip of the micropipette by 30° (see Figure 3c). In this way, the tip can be horizontal after being installed on the robot. In the same way, we also bend the holding micropipette by the same degree before mounting it on the right arm. In this way, the axes of the cell, micropipette electrode, and holding pipette are in a horizontal state during the experiment.

Then, the micropipette was backfilled with 20 μL electrode liquid. Before the measurement, the cell culture medium was replaced by extracellular fluid (g/L: HEPES 0.6911, C_16_H_18_N_2_O_4_S 0.05, NaHCO_3_ 2.2504, NaCl 1.7532, C_21_H_39_N_7_O_12_ 0.06, M199 9.5, pH 7.0~7.2) for electrical signal recording. A silver electrode wire with a diameter of 0.2 mm was inserted into the micropipette to form the micropipette electrode. One section of the micropipette electrode was immersed in the extracellular fluid, and the ground wire connected at the other end was directly placed in the extracellular fluid, thus forming a complete circuit.

The oocytes we used in the experiment were obtained using the following methods. We collected pig ovaries from the local slaughterhouse and used a sterile 10mL syringe with an 18-gauge needle to aspirate ovarian follicles with a diameter of 3–6 mm to obtain cumulus–oocyte complexes (COCs). At this time, there are many tissue impurities in the COCs liquid. To obtain clean COCs, we poured them into Tyrode’s lactate (TL)–Hepes-PVA (polyvinyl alcohol, 0.1%) for washing. After washing, we waited for their precipitation for 20 min and then poured out the supernatant to wash and precipitate again, repeating three times. Then, 20~40 COCs were picked up and transferred to 100 μL mature medium (TCM-199 supplemented with 15% FBS, 10 ng/mL EGF, 10% porcine follicular fluid, 10 IU/mL of eCG, 5 IU/mL of hCG, 0.8 mM L-glutamine, and 0.05 mg/mL gentamicin) for 42 h at 38.5 °C, 5% CO_2_, and saturated humidity. At the MII stage, we selected target oocytes that had a clear perivitelline space, integrated cell membrane, and a visible polar body (PB1) (Figure 3d).

### 2.3. Resistance Analysis for Micropipette Electrode Out of the Cell

It is well known that when the glass micropipette is filled with electrolyte solution inside and outside, and an electromotive force is applied, the glass micropipette can form a stable resistance.

**Figure 4:**
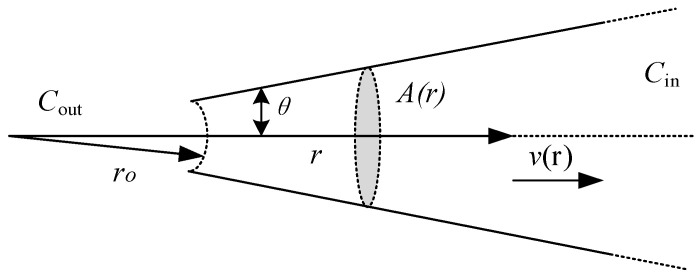
Schematic diagram of micropipette structure.

The tip diameter used in our experiment was ~2 μm pipette, whose geometric structure is similar to an infinite cone (Figure 4). We chose KCl solution as the internal solution of the electrode and assumed that KCl diffusion, just like in isotropic homogeneous media, increases the KCl flux due to a large amount of flow:

(1) $$\frac{\partial C}{\partial t}=D\frac{\partial^{2}C}{\partial r^{2}}+\frac{2D}{r}\frac{\partial C}{\partial r}-v\left(r\right)\frac{\partial C}{\partial r}$$

where *C* = KCl concentration (moles/liter), *D* = KCl diffusion coefficient (cm^2^/s), *r* = distance from origin of cone (cm), and *v*(*r*) = fluid velocity at *r* (cm/s).

It is assumed that the fluid velocity is the same on any given cross-section (“plug” flow), and the velocity passes through the origin of the cone (which allows a one-dimensional solution in a spherical coordinate system). For a given flow, the speed of each cross-section will be inversely proportional to the square of the radius of that cross-section (Equation (2))

(2) $$v\left(r\right)=\left(k/r^{2}\right)\left(P_{out}-P_{in}\right)$$

where *k* = a constant containing geometrical terms and pipet hydraulic resistance, *P_in_* = pressure applied to the inside of the pipet (mm Hg), and *P_out_* = outside pressure (mm Hg).

We can calculate the total pipette resistance by integrating the conductivity on the length of the pipette.

(3) $$R=\int_{r_{o}}^{\infty }\frac{1}{K_{sp}A\left(r\right)}dr$$

where *R* = total pipet resistance (Ω), *r_o_* = distance of the tip from the origin of the cone (cm), and *A*(*r*) = cross-sectional area at *r* (cm^2^).

The electrode resistance is affected by the solution concentration. When the solution concentration decreases, the measured electrode resistance increases. When the solution concentration (less than the solubility of the solution itself) increases, the measured electrode resistance decreases.

**Figure 5:**
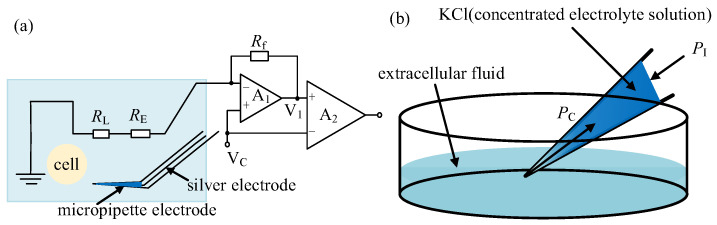
Resistance analysis for micropipette electrode out of the cell: (**a**) The measurement micropipette electrode circuit; (**b**) Ion concentration distribution in micropipette electrode.

When the micropipette electrode is out of the cell, the measurement micropipette electrode circuit is shown in Figure 5a. The micropipette electrode measurement resistance *R*_M_ is composed of the silver wire electrode resistance *R*_E_, the solution resistance *R*_L_, and the resistance *R*_I_ caused by the concentration gradient field of the electrode solution and the extracellular solution near the micropipette electrode opening, namely

(4) $$R_{M}=R_{E}+R_{L}+R_{I}$$

Among them, *R*_E_ and *R*_L_ can be considered to be constant when the volume of the liquid enters or comes out of the micropipette is negligible in comparison to the whole volume of liquid inside the micropipette and the environment. For *R*_I_ in Equation (4), the ion concentration gradient field corresponding to *R*_I_ outside of the cell is formed by the combined action of the following two factors: capillary pressure *P*_C_ between the tube wall and the liquid in the electrode and the injection pressure *P*_I_ in the microtubule, as shown in Figure 5b. When *P*_I_ is small, capillary pressure *P*_C_ will press extracellular fluid (dilute electrolyte solution) into the electrode to form a stable ion concentration gradient and *P*_I_ value. When the *P*_I_ increases, the ion concentration distribution at the tip will shift to the opening, resulting in an increase in the concentration of KCl at the tip, which will lead to a decrease in the resistance of the electrode. When *P*_I_ is larger enough, the concentration gradient area will be pushed out of the micropipette opening. In that case, *R*_M_ will reach a stable state with a further increase in *P*_I_ if the deposition volume of the solution into the environment is negligible in comparison to the whole volume of solution inside the micropipette.

In summary, a “downslope” section of measurement resistance of the micropipette can be obtained with an increase in *P*_I_. When the micropipette enters intracellular space, the intracellular pressure leads to a shift of concentration gradient inside the micropipette and subsequently changes the measured resistance of the micropipette electrode. Thus, the variation of the measured electrode resistance can be utilized to measure the intracellular pressure.

### 2.4. Prepare the Required Electrode Internal Liquid and Measure the Working Range of the Probe Micropipette Electrode

As aforementioned, the variation in the measured resistance of the micropipette electrode can be utilized to measure intracellular pressure. In the experiment, we chose KCl solution as the electrode solution. To guarantee measurement accuracy, an appropriate concentration of the solution producing a steep downslope in the *R*_M_-*P*_I_ relationship is preferred because the *R*_M_ is more sensitive to the variation of *P*_I_ in that case, which improves the measurement accuracy of *P*_I_. An increase in KCl concentration increases the height of the downslope, according to the analysis in Section 2.3. However, a too-large KCl concentration will increase the crystallization of KCl molecules and increase the blocking issues of the micropipette opening. Thus, an appropriate KCl concentration needs to be determined through tests.

**Figure 6:**
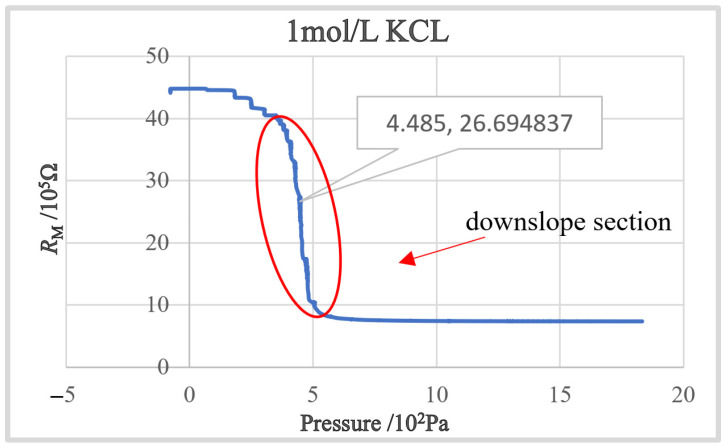
The *R*_M_-*P*_I_ curve with a downslope section.

The concentration gap of the KCl solution starts to increase from 0.1 mol (pure water) to 3 mol with an interval of 0.1 mol until the experiment is stopped due to the crystal blocking of the nozzle due to the excessive concentration of the solution. According to testing results, we found that the decreasing speed of *R*_M_ increases in the slope area as the concentration of KCl solution increases at the beginning. After the concentration is larger than 1 mol, the blocking incidences start to occur, and its occurrence rate grows as the concentration increases. Therefore, in the following experiment, we chose KCl with a concentration of 1 mol/L as the electrode internal solution. Figure 6 shows the obtained *R*_M_-*P*_I_ curve. It can be found that a steep downslope section, which is the ideal working section for intracellular pressure measurement, exists in the obtained *R*_M_-*P*_I_ curve.

### 2.5. Intracellular Pressure Measurement Based on Electrode Resistance Model

**Figure 7:**
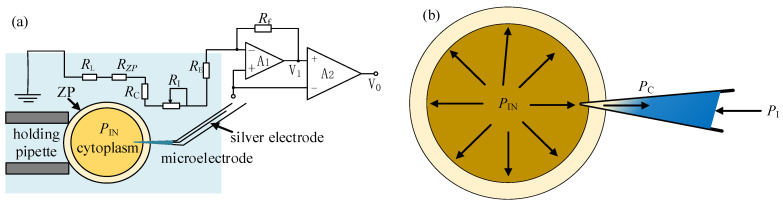
Electrode resistance model for measuring intracellular pressure: (**a**) The resistance model of micropipette electrode; (**b**) The ion concentration distribution in the micropipette electrode after cell penetration.

To measure the intracellular pressure, the measurement resistance of the micropipette electrode is modeled, as shown in Figure 7a. When the micropipette electrode enters the cell, the zona pellucida resistance *R*_ZP_ and the cytoplasmic resistance *R*_C_ also become part of the measurement resistance *R*_M_, namely

(5) $$R_{M}=R_{E}+R_{L}+R_{I}+R_{C}+R_{\mathrm{ZP}}$$

Among them, *R*_E_, *R*_L_, and *R*_ZP_ can be regarded as constants in the experiment. The cytoplasmic resistance *R*_C_ can be regarded as constant on the premise that the micropipette electrode is sealed with the ZP and the amount of injected material is far less than the cytoplasmic volume during the puncture process. After the micropipette electrode is inserted into the cell before the intracellular pressure is released, the ion concentration gradient field corresponding to *R*_I_ is formed by the joint action of the intracellular pressure *P*_In_, the injection pressure *P*_I_, and the capillary pressure *P*_C_, as shown in Figure 7b. Adjusting the *P*_I_, *R*_I,_ and *R*_M_ will also change accordingly. When the applied *P*_I_ remains unchanged and the change amplitude of *R*_M_ within a certain period of time does not exceed 3% of the current value, it is considered to have reached a quasi-stable state. Record the *P*_I1_ at this time and retreat the micropipette electrode. Then, the micropipette electrode enters intracellular space again after the release of intracellular pressure. The ion concentration gradient field corresponding to *R*_I_ is formed again by injection pressure *P*_I_ and capillary pressure *P*_C_. Then, a new stable state of *R*_M_ similar to the former one before intracellular pressure release can be achieved through adjustment of *P*_I_ again. The difference between the two injection pressures *P*_I_ forming the stable states of *R*_M_ before and after the release of intracellular pressure separately can be considered as the released intracellular pressure value.

### 2.6. Robotic Measurement Procedure of Intracellular Pressure Measurement

**Figure 8:**
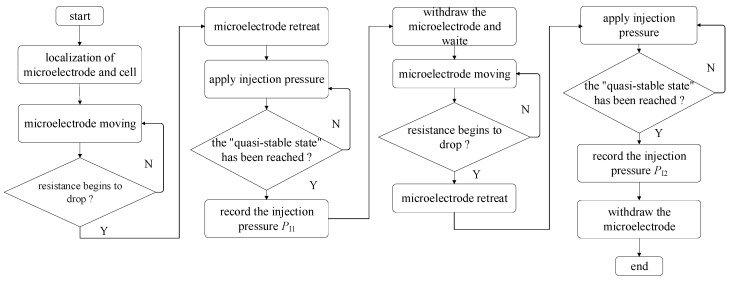
Flow chart for robotic measurement of intracellular pressure.

A robotic measurement procedure of intracellular pressure was developed based on the above work. Figure 8 summarizes the robotic measurement procedure.

Before measurement, the micropipette electrode and the holding micropipette were moved into the field of vision manually. Then, one oocyte was put into the field of view. The system automatically focuses and 3D localizes the micropipette electrode, holding micropipette, and the target oocyte [19,20], corresponding to the frame “localization of microelectrode and cell” in Figure 8. Then, the system controlled the holding micropipette to approach the target oocyte and immobilize it with aspiration pressure [21].

Before cell membrane (zona pellucid (ZP) for porcine oocyte in this paper) penetration, the injection pressure inside the micropipette electrode was adjusted to make the resistance of the micropipette electrode within the working range (downslope section). After that, the micropipette electrode was controlled to penetrate the cell membrane along the central axis of the holding micropipette, and the system automatically detected the resistance of the micropipette electrode, corresponding to the frame “microelectrode moving” in Figure 8. Once the micropipette electrode contacts the cell, the detected resistance will start to rise, and the resistance will continue to rise as the micropipette electrode advances. When the detected micropipette electrode resistance begins to decrease, which means the micropipette has entered the intracellular space, the system assumes that the cell membrane has been punctured, corresponding to the frame “resistance begins to drop?” in Figure 8. To reduce the squeezing of micropipette electrode cells, which usually generates a false larger intracellular pressure, the system controls the micropipette electrode to retreat until the cytoplasm contour recovers its sphere shape, which means the deformation resulting from cell membrane penetration fully recovers, corresponding to the frame “microelectrode retreat” in Figure 8. The contour detection method for cytoplasm has been reported in our previous research [22].

After the cell membrane penetrates, the system increases *P*_I_, and *R*_M_ decreases accordingly, corresponding to the frame “apply injection pressure” in Figure 8. This is because a 1 mol/L KCl solution is a concentrated electrolyte solution compared to the intracellular fluid. When the applied *P*_I_ remains unchanged and the amplitude of *R*_M_ change within 5 s does not exceed 3% of the current value, it is considered to have reached a quasi-stable state, corresponding to the frame “the quasi-stable state has been reached?” in Figure 8. *P*_I1_ was recorded at this time, and the micropipette electrode was withdrawn from the cell along the axial direction of the holding micropipette and waited for 60 s to release the intracellular pressure, corresponding to frames “record the injection pressure *P*_I1_” and “withdraw the microelectrode and wait” in Figure 8. Then, the system controlled the holding pressure of the holding micropipette to first spit and then hold the cell again. In this way, the cell rotated by some degrees relative to the original position. Then, the micropipette electrode entered the cell again along the axis direction, retreated to let the cell recover its shape, increased the injection pressure again to reach the quasi-stable state, and recorded the required injection pressure *P*_I2_ at this time. The intracellular pressure of cells is then determined by the difference in injection pressure before and after the release of intracellular pressure. The corresponding *P*_I2_ is considered valid only when the difference in resistance values between two quasi-stable states does not exceed 100 Ω, which means that the measurement results can be adopted. We have established a flowchart for the robotic measurement of intracellular pressure (see Figure 8).

The reason why we make the cell rotate relative to the original position before entering the cell again is given as follows. The micropipette electrode usually releases a small amount of electrode fluid (concentrated electrolyte solution) around the point it stays after the first puncture. Due to the poor diffusion of the KCl solution inside the cell in comparison to that outside the cell, there may still be some KCL solution around the previous point. If the micropipette tip stays at the same position after the second entry, the existence of the left KCl solution may weaken the concentration gradient and the variation of *R*_M_ along *P*_I_, increasing the difficulty of obtaining *P*_I2_. Therefore, cell rotation before the second entry is required to make the position it stays inside the cell different from the first one. This ensures that the micropipette electrode tip is filled with KCl solution on the inner side and pure intracellular liquid on the outside during the two penetration processes of the cell.

## 3. Results

### 3.1. Intracellular Pressure Measurement Results

We used porcine oocytes in the MII period as experimental samples. The extracellular fluid was the cell operating fluid (H199) that could keep the oocytes active in vitro. The micropipette electrode was filled with 1 mol/L KCl solution. All experimental animal protocols were approved by the Institutional Animal 129 Care and Use Committee of Nankai University and carried out following the national 130 ethical guidelines for laboratory animals (permission number SYKX 2019-0001).

**Figure 9:**
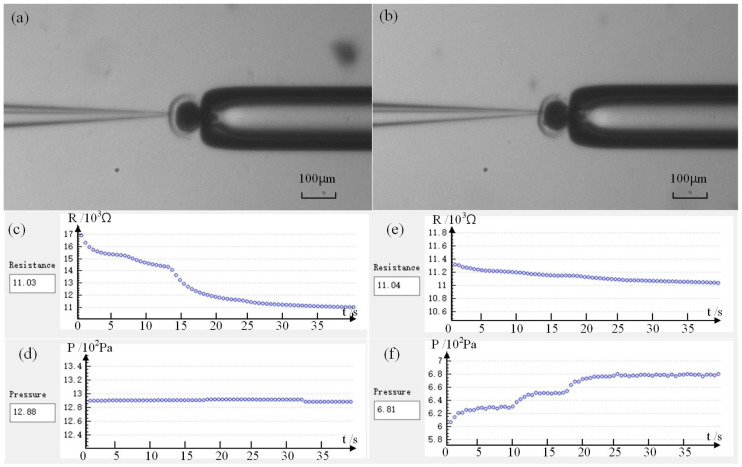
Intracellular pressure measurement results: (**a**) Micropipette electrode penetrates cell before releasing intracellular pressure; (**b**) Micropipette electrode penetrates cells after the release of intracellular pressure; (**c**,**d**) Resistance value and corresponding pressure in the first quasi-stable state; (**e**,**f**) Resistance value and corresponding pressure in the second quasi-stable state.

Figure 9a shows the first penetration of the micropipette electrode into the cell, while Figure 9b shows the second penetration after the release of intracellular pressure. Figure 9c–f shows the detection resistance and injection pressure in our experiment, respectively. Figure 9c,d shows data sampling after the first puncture. Figure 9e,f shows data sampling after the intracellular pressure is released and then inserted again. From Figure 9, we can see that after the micropipette electrode is inserted into the cell for the first time and the detection resistance reaches the quasi-stable state, the corresponding injection pressure *P*_I1_ = 1288 Pa. The corresponding injection pressure *P*_I2_ = 681 Pa after the second insertion and the detection resistance is stable. Based on the above research, we can calculate the intracellular pressure of this oocyte as *P*_in_ = *P*_I1_ − *P*_I2_ = 607 Pa. According to statistics, the intracellular pressure of porcine oocytes we measured is 200~700 Pa with an average value of 500 ± 50 Pa (n = 20), which is basically consistent with the numerical range reported in the relevant literature [13,14]. This consistency proves the effectiveness and feasibility of our measurement method.

Finally, after one week of practice, the average measurement speed of the proposed method is 8 ± 2 cells/h. During the experiment, it is usually necessary to change a new micropipette after measuring a cell. This is because multiple punctures into the cells will cause some cytoplasm to adhere around the micropipette nozzle, which will lead to unstable detection resistance. Actually, the replacement and preparation of new micropipette costs most of the operation time for each cell (about 5 min). The measurement success rate is 50% (n/m) at present. With this measurement efficiency, the system is expected to measure about 20~40 cells a day, totally comparable to that of the method using commercial devices [15], which are usually able to measure 10 cells per day after two weeks of practice. We compare the operating time of our method with that of using commercial equipment methods in Table 1. Our method is fast enough to measure intracellular pressure compared to previous methods.

### 3.2. Leakage Test for Intracellular Pressure during the Measurement Process

During the measurement process of intracellular pressure, the ZP and outside wall of the micropipette need to be well sealed to avoid leakage of intracellular pressure. Otherwise, the leakage of intracellular pressure leads to a falsely smaller value of measured intracellular pressure than its original one. In this part, the fluorescent dye solution was backfilled into the micropipette to detect possible leakage of intracell pressure during measurement.

**Figure 10:**
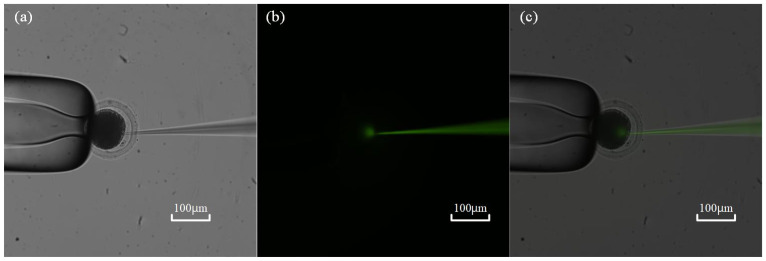
Leakage testing of intracellular pressure: (**a**) Micropipette electrode penetration into cells under bright field; (**b**) Observation of fluorescence diffusion within cells under fluorescence field; (**c**) Synthesis of two images under bright and fluorescent fields.

First, a fluorescent dye (HTPS, 5 mM) solution was backfilled into the micropipette before measurement. After ZP penetration, a positive pressure pulse was exerted inside the micropipette to deposit about 2 pL volume of solution, only about 0.25% of the total volume of the oocyte calibrated by the method mentioned in previous research [11]. Then, the field of view switches to the fluorescent field to check the diffusion scope of the fluorescent dye. If any fluorescent dye diffuses outside of the cell through the wound, it means that the seal between the micropipette and the ZP has failed, and leakage of intracellular pressure occurs during measurement. According to the experimental results on 10 oocytes, we found no leakage of intracellular pressure during the measurement process (see Figure 10), which guarantees the measurement accuracy of the intracellular pressure using our method.

### 3.3. Repeatability Validation Experiment

Unfortunately, as an invasive measurement method, our method is not able to measure the intracellular pressure of one cell more than once because the intracellular pressure leaks from the wound after the first measurement. Thus, the repeatability of our measurement method cannot be evaluated through comparison among the obtained intracellular pressure multiple times for the same cell. However, as a measurement method relying on the *R*_M_-*P*_I_ relationship, the repeatability of our measurement method can be indirectly evaluated through the repeatability of the obtained *R*_M_-*P*_I_ curves. Due to the aforementioned poor diffusion rate of the released KCl solution inside the cell, the local concentration gradient at the opening of the micropipette and the obtained *R*_M_-*P*_I_ curves can be easily varied because of the KCl solution released from the micropipette during the test. Thus, the test was conducted in H199 fluid outside the cell, where the diffusion of the KCl solution is much faster. Further, compared to the volume of KCl injected by the micropipette electrode, the volume of H199 fluid can be assumed to be infinite. Thus, the environment can be considered stable in multiple experiments.

**Figure 11:**
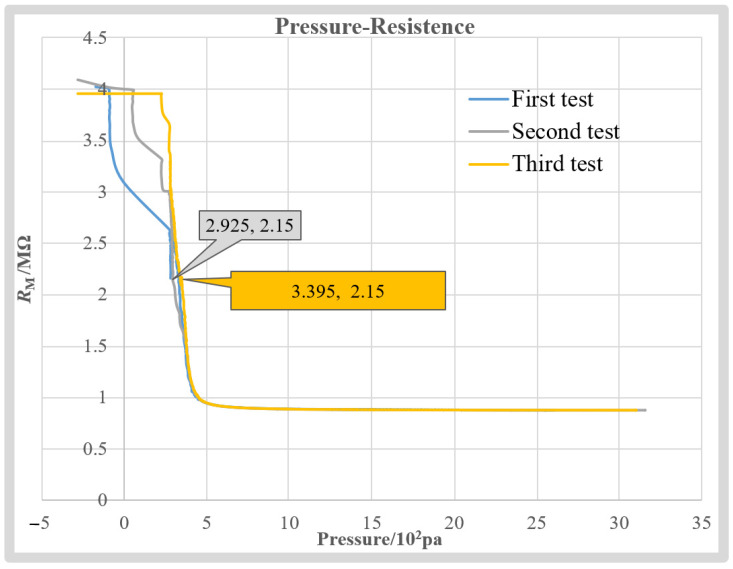
Repeatability validation experiments.

After the test starts, the micropipette electrode is immersed in the H199 solution. Then, injection pressure inside micropipette *P*_I_ is back-and-forth adjusted to record the corresponding measurement resistance, *R*_M_, repeated three times. The obtained *R*_M_-*P*_I_ curves are summarized in Figure 11. We can see that when the recorded pressure is greater than 300 Pa, the obtained *R*_M_-*P*_I_ curves almost coincide with each other. When the resistance value is the same, the pressure value error is less than 47 Pa. The average error is 22 Pa, which is only 4.4% of the average measured intracellular pressure. It is worth noting that the consistency of the three curves reduces when *P*_I_ is less than 300 Pa. This is mainly because the openness of the valve to adjust the *P*_I_ is almost closed when the output pressure is less than 300 Pa, which easily produces an unstable air flow, resulting in unsteady *R*_M_. Fortunately, the diameter used in this article is ~2 μm. To keep the resistance value of the micropipette electrode within the working range (downslope section) and prevent the tip from being blocked in the process of penetrating the cell, the system will give the micropipette electrode a positive pressure greater than 300 Pa before the penetration. Therefore, *R*_M_ disturbances below 300 Pa have no significant impact on our measurement results. The good repeatability of the *R*_M_-*P*_I_ curve obtained ensures the repeatability of the proposed intracellular pressure measurement method.

### 3.4. Cell Survival Rate Testing Results after Measurement

**Figure 12:**
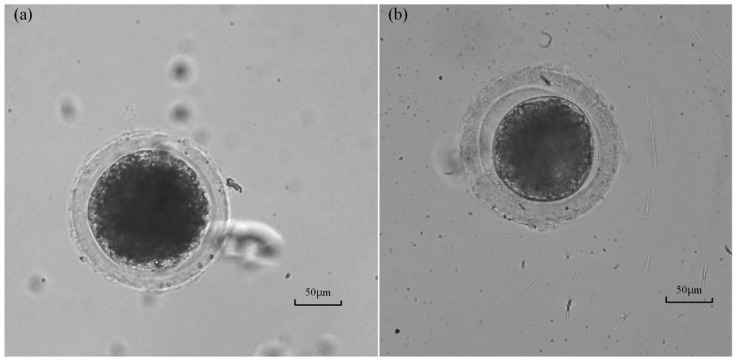
Oocyte images after 2 h of culturing after the measurement: (**a**) The oocyte with part of a broken membrane which may be a dead oocyte. (**b**) Alive oocyte with an integrated cytoplasm membrane.

The survival rate of the measured oocyte was tested after intracellular pressure measurement to evaluate the harm caused by the proposed method to the oocyte viability. The operated oocyte is considered to survive if a clear and integrated cytoplasm membrane exists (see Figure 12a), which means the wound has healed after 2 h of culture. If part of the cytoplasm membrane still breaks at that time (see Figure 12b), which means the wound is still open, the viability of the oocyte is considered to be damaged, and the oocyte may be dead. According to the observation results, only two oocytes with a broken cytoplasm membrane were found from the twenty oocytes with successful measurement. The above results demonstrate that the survival rate of the oocyte measured by the proposed method is up to 90% (18/20), proving limited harm to cell viability. This is very important for biological applications requiring alive oocytes or embryos, such as oocyte or embryo microinjections. For these applications, the in situ measurement of intracellular pressure can be achieved using our method before operations.

The use of the oil injection method to measure intracellular pressure is also an invasive measurement method [11]. However, as is well known, mineral oil is a harmful substance to cells, and cells measured using this method are almost unable to maintain activity. Table 1 compares our method with the oil injection method in terms of cell survival rate after measuring intracellular pressure. Our method causes less damage to cells compared to the oil injection method.

## 4. Discussion

Our method can be conducted using a common microoperation system with the measurement ability of the micropipette electrode resistance. Because it does not involve expensive specific commercial measurement devices, which usually cost USD 80,000–90,000 [23], the proposed system is expected to have more widespread applications. Through comparing the required *P*_I_ making *R*_M_ reach a quasi-stable state before and after the release of the intracellular pressure, the influences of the cytoplasm and ZP on the *R*_M_ are excluded, which may improve the measurement accuracy of intracellular pressure in comparison to the method based on commercial devices [15].

Although our system is only able to measure one cell at one time at present, it is highly expected to achieve robotic measurement of batch cells in the future. We proposed a robotic enucleation method [20] for batch oocytes in the previous study. In that method, the system automatically scanned the entire area of oocytes and established a global map containing all the target oocytes before operation. With the global localization of the target oocytes, the system was able to pick up the target cell with the holding micropipette, move the cell to another place with movement of the microstage, and then operate it with the injection micropipette one by one. Further, the pancreatin solution has been proven to be able to clear the inner surface of the micropipette and remove the adherent cell materials on it [24]. With a droplet of pancreatin solution to wash the micropipette after measurement on each cell, the replacement of the micropipette can be saved. Similar functions can be integrated into our system for the robotic measurement of batch cells in the future. After that, the time cost in search of each cell and replacement of the micropipette can be saved, which will significantly accelerate the operation speed of our method.

At present, the success rate of the proposed system is about 50%. The two main reasons for the measurement failures are the blockage of the micropipette electrode opening and failures of quasi-stable state formation after ZP penetration. Apparently, the increase in the diameter of the micropipette electrode and the decrease in the concentration of KCl concentration are both able to reduce the blockage of the micropipette opening. However, a micropipette tip with a larger opening increases the cut size generated during penetration and subsequently increases the leakage incidences of the intracellular pressure. Moreover, the decrease in KCl concentration may further reduce the concentration gradient at the micropipette opening after ZP penetration, which finally reduces the formation success rate of the quasi-stable state of *R*_M_. Sharpening the tip with a tilt opening may be a better way to reduce the blockage in the future, as it can increase the opening area of the micropipette and does not enlarge the wound. Increasing the diffusion speed of the solution may increase the formation success rate of the quasi-stable state. Thus, a compound solution with a diffusion speed higher than KCl is expected to replace the KCl solution in the future for the improvement of the formation success rate of the quasi-stable state of *R*_M_. In addition, after completing the experiment, some KCl solution will be left in the cell, which may affect the osmotic pressure of the cell. Therefore, we speculate that the left KCL has an impact on cell viability. However, from our conclusion in Section 3.4, we can see that the cell survival rate reaches 90%, so we can infer that the impact of left KCL on the cell survival rate is very small.

Additionally, it has been reported that the speed at which the micropipette penetrates cells can have an impact on cell deformation and damage, and optimizing the penetration speed can reduce the damage caused by micropipette penetration to cells [25]. This inspires the potential improvement of the work in this paper. Currently, in our experiment, the micropipette electrode penetration into cells is at a constant speed of 10 μm/s. In the future, we can control the penetration speed to reduce cell damage.

## 5. Conclusions

In this paper, a method of intracellular pressure measurement based on a micropipette electrode circuit model is proposed. We first introduced the fabrication method of the micropipette electrode used in the experiment. Secondly, according to the change principle of electrode detection resistance, we established the circuit model of the micropipette electrode outside the cell and carried out the extracellular injection experiment to determine the appropriate concentration of the electrode liquid used in the experiment. Then, we established the circuit model of the micropipette electrode inside the cell. Finally, a robotic measurement procedure was established based on the above work. We carried out our intracellular pressure measurement experiment on the patch clamp system in our laboratory and obtained the intracellular pressure of porcine oocytes (200~700 Pa), which is basically consistent with the value reported in the relevant literature. The effectiveness and feasibility of our proposed method were validated through experiments. Although our experiment was implemented on a patch clamp system, this method is easy to generalize to conventional micropipette electrode resistance measurement systems, so it does not require expensive systems and helps achieve in situ detection.

## Figures and Tables

**Table 1:** Comparison between our method and previous work.

Comparison	Our Method	Commercial Equipment	Oil Injection Method
number of cells operated	20~40 cells/day	10 cells/day	20 cells/day
practice time cell survival rate	1 week	2 weeks	/
	90%	/	0%

## Data Availability

The raw data supporting the conclusions of this article will be made available by the authors, without undue reservation.
